# Modulation of energy and protein supplies in sequential feeding in laying hens

**DOI:** 10.1017/S1751731114002092

**Published:** 2014-09-05

**Authors:** M. Traineau, I. Bouvarel, C. Mulsant, L. Roffidal, C. Launay, P. Lescoat

**Affiliations:** 1 INRA, URA (UR83), F-37380 Nouzilly, France; 2 INZO, Service R&D Pondeuses, Rue de l'Eglise, CS 90019, 02400 Chierry, France; 3 ITAVI, F-37380 Nouzilly, France; 4 AgroParisTech, UMR1048 SADAPT 16 rue Claude Bernard, 75005 Paris, France

**Keywords:** laying hens, sequential feeding, energy, protein, environmental conditions

## Abstract

Sequential feeding (SF) consists of splitting energy (E) and protein/calcium (P) fractions temporally, improving the feed conversion ratio (FCR) of hens compared with a continuous distribution during the day. In a previous study, the E fraction (with a low level of protein) was provided in the morning, whereas the P fraction (with low level of energy) was given in the afternoon. However, there is no clear evidence that a requirement in energy or proteins is connected to these distribution sequences, whereas the requirement for calcium is known to be required in the afternoon. To evaluate the effects on performances of the modulation of energy and protein supplies in SF, five different sequential treatments were offered: E0P0/E0P0; E+P+/E−P−; E+P−/E−P+; E0P+/E0P− and E+P0/E−P0 where E+ represents a high energy level, E0 a moderate one and E− a low one (with the same meaning for P regarding protein supply). Afternoon fractions were provided with particulate calcium. A total of 168 Hendrix hens were housed in individual cages from 20 to 39 weeks of age in two environmentally contrasted rooms. Feed intake in the morning and afternoon fractions, egg production, egg weight, BW and weight of digestive organs were recorded. No diet effect was observed concerning feed intake, egg production and BW. These results suggested that hens are not able to fit their feed intake on energy or protein level of fractions within half-day duration, whereas at the day scale same protein and energy intakes were observed. Moreover, the time of nutrient distribution in feeding did not seem to have an impact on birds’ performances. These studies have also demonstrated that, despite strong environmental pressure, the hens with SF had attenuated performance but continue to produce eggs.

## Implications

Improving laying hen performance may be achieved by maximizing nutrient utilization from current feedstuff. Supply of various energy and protein levels in sequential feeding could reduce the amount of food ingested while maintaining an equivalent level of production. Knowledge of the specific needs in energy and protein during the day will allow optimizing daily intake. This study can increase the robustness of the system to make it usable on a field scale and to ensure the relevant nutrient supply sequence, although time of supply does not seem to be of strong impact, except for Ca.

## Introduction

Several studies on Sequential Feeding (SF) have yielded good results through a meaningful improvement of the feed conversion ratio (FCR) while maintaining similar performances compared with hens fed classically with continuous diets. The morning fractions were rich in energy, whereas the afternoon ones were rich in proteins &&and calcium (Umar Faruk *et al.*, 2010 and 2011; Traineau *et al.*, 2013). However, in these SF studies, hens fed with fractions with higher energy content in the morning than in the afternoon ate the same quantity of energy than the hens fed with the control diet on a day scale. In the morning, lower feed intake might be because of the regulation on energy in the diet (Gunawardana *et al.*, 2009; Perez-Bonilla *et al.*, 2012). Increased apparent metabolizable energy content of the diet reduced feed intake to maintain an optimal level of energy ingested (Valkonen *et al.*, 2008), supporting the assumption that feed intake depends on the level of energy in the diet at the day scale. In SF, even if the level of energy is high in the morning and low in the afternoon, hens seem to be able to regulate their feed intake to have a same quantity of energy intake compared with continuous diet. Moreover, reduced feed intake, while maintaining identical laying performance, induced significant improvement of FCR (Grobas *et al.*, 1999; Valkonen *et al.*, 2008). These results might support the improvement in FCR for hens fed sequentially with a high-energy fraction in the morning and a high protein one in the afternoon. However, it is difficult to demonstrate that hens need energy mostly in the morning and protein in the afternoon according to their specific requirements connected with the egg production cycle, whereas several studies have shown that hens express a specific appetite for calcium in the afternoon for eggshell formation (Mongin and Sauveur, 1974; Chah and Moran, 1985). In all studies on SF, calcium was provided in the afternoon fraction in coarse form of calcium carbonate (Traineau *et al.*, 2013; Batonon *et al.*, 2014). To improve SF system robustness and to implement it on a commercial scale, it has to be more flexible. Traineau *et al.* (2013) showed that the energy fraction could be formulated with wheat or corn or with a mix of these two cereals. These diets lead to intermediate results between whole wheat in SF and usual continuous diet. This substitution between these cereals gives more flexibility for diet formulation compared with previous studies on SF (Umar Faruk *et al.*, 2010). Regarding nutrient supply, Penz and Jensen (1991) showed that a feeding timetable could be a solution when using low-protein diets in layer diets while maintaining similar laying performances. When hens were fed a ‘high-protein’ diet (16% CP) from 0400 to 0800 h and from 1400 to 2000 h, and a ‘low-protein’ diet (13% CP) from 0800 to 1400 h, no difference in performance compared with control hens (16% CP) was observed. However, hens fed continuously the ‘low-protein diet’ (13% CP) or fed the ‘low-protein’ diet from 0400 to 0800 h and from 1400 to 2000 h, and the ‘high-protein’ diet from 0800 to 1400 h, laid lower egg weights than the control group. This study supported that a provision of protein at given times influences the performances and has strengthened the hypothesis that hens need more protein after oviposition to meet the protein requirements for the synthesis of the albumen even though secretion might not be fully related to protein synthesis. However, Keshavarz (1998) had a similar experimental design (positive control 16% CP, negative control 13% CP, and two sequential diets combining 13% and 16% CP fractions), and concluded that optimum performance may be expected as long as the daily protein intake is adequate and satisfies the daily needs of laying hens. Therefore, additional experiments are required to further investigate SF. In addition, laying hens’ performances not only depend on feed intake but also on other environmental effects. The cage size had an important effect on the laying performances by reducing the laying rate (Cunningham *et al.*, 1988), and small cages induce more cracked and dirty eggs (Abrahamsson and Tauson, 1997). In addition, apparition of parasites in the rearing room could decrease the hens’ performances. Red mites’ pressure leads to decrease in feed consumption and reduce eggs’ quality and production (Lesna *et al.*, 2009).

The first aim of this study was, by modulating the energy and protein fractions and nutritional contents in SF for laying hens, to determine their impacts on intake, growth and production of hens because of the sequential supply of nutrients. The second aim was to define whether the ranking of the diet was the same event if hens are reared in different conditions. This study should therefore help to determine the nutritional needs of the hens at different times of the day.

## Material and methods

### Birds and housing

A total of 140 Hendrix layer hens were housed in 2 two-tier batteries having individual cages equipped with a nipple drinker. For the duration of the experiment, the temperature was maintained in both rooms between 18°C and 22°C. All hens had individual feeders and have been randomly assigned in battery cages. The birds were habituated to SF with a mix of whole wheat and a balancer from weeks 16 to 18. The photoperiod was 12L : 12D at week 16 and reached 16L : 8D at week 18 and remained at this level until the end of experiment. Lights were on at week 16 from 0500 to 1700 h and from 0400 to 2000 h from weeks 18 to 39.

The experimental period ran from weeks 19 to 39. Birds were housed in two rooms within the same building and kept in individual cages from weeks 16 to 39. Birds were divided into five groups of 12 birds in the first room, and into five groups of 16 birds in the second room. The difference between the two rooms was mainly related to the size of the cages: in the first room cages were 45 cm width×62 cm depth×80 cm height, and in the second room 25 cm width×48 cm depth×48 cm height. Moreover, the second room had an environmental pressure because of red mites. This room was progressively infected with red mites for the duration of the experimental period, which altered whole performance of hens, despite treatments against mites.

### Experimental treatments

Diets fed during the experimental period are described in Table 1. Dietary contents of previous experiments on SF in our lab were based on the utilization of two different cereals in the morning fraction (wheat and maize; Traineau *et al.*, 2013); nevertheless, the present study is focused on the nutritional values of the offered diets in SF. Diets were distributed sequentially. Birds were offered 62.5 g in the morning and beginning of the afternoon (0830 to 1530 h), and 62.5 g in the late afternoon and early morning (1530 to 0800 h and 0400 to 0830 h), 125 g/day being 105% of the recommended diet supply. For all diets, calcium is provided in coarse form, in the fraction distributed in the afternoon. No diet was formulated with a low-energy level (E−) in the morning fraction. This fraction was systematically less consumed than the afternoon one for every previous study, inducing overall shortage in ME intake. To modulate the daily intakes’ dynamic of energy and protein, five sequential diets were offered: E0P0/E0P0 (2601 kcal/kg; 16.5 CP/2601kcal/kg; 16.5 CP); E+P+/E−P− (2901 kcal/kg; 19.5 CP; 2303 kal/kg; 13.5 CP); E+P−/E−P+ (2905 kcal/kg; 13.5 CP/2302 kcal/kg; 19.6 CP); E0P+/E0P− (2599 kcal/kg; 19.6 CP/2597 kcal/kg; 13.6 CP) and E+P0/E−P0 (2903 kcal/kg; 16.4 CP/2302 kcal/kg; 16.5 CP). Diets were formulated according to INRA recommendations, and proposed nutritional contents were based on the hypothesis of 50/50 intakes of the morning and afternoon fractions (Sauvant *et al.*, 2004). Water was given *ad libitum* throughout the experimental period.

**Table 1:** Composition of experimental diets given to laying hens between 19 and 39 weeks of age with a combination of 62.5 g of morning fraction between 0830 and 1530 h and 62.5 g of afternoon fraction between 1530 and 0830 h (no light between 2000 and 0400 h)

	E0P0/E0P0		E+P+/E−P−		E+P−/E−P+		E0P+/E0P−		E+P0/E−P0	
	E0P0 morning	E0P0 Ca+ afternoon	E+P+ morning	E−P− Ca+ afternoon	E+P− morning	E-P+ Ca+ afternoon	E0P+ morning	E0P− Ca+ afternoon	E+P0 morning	E−P0 Ca+ afternoon
Ingredient (%)										
Corn	31.1	30	33	28	39.7	23.2	27	33.1	38	25
Wheat	30	29	34.6	28.7	39	22	27.9	33	36.3	26
Soybean meal	9.5	17	17	4	6	19	16.5	13.5	13	11
Rapeseed meal	5		5	5	5	5	5		5	5
Sunflower meal	10		3.7	10	0.3	9.1	10		4.8	10
Wheat bran	10			4.5	7		10			2
Calcium carbonate	1.57	15.36		15.35		15.24		15.38		15.3
Corn gluten		4.8	3.7	1.4		3.4	0.8	1.3	0.2	2.7
Dicalcium phosphate	1.01	1.34	1.15	1.19	1.1	1.23	0.97	1.35	1.17	1.21
Soybean oil	0.5	1.2	0.5	0.5	0.5	0.5	0.5	1.1	0.5	0.5
Premix^1^	1	1	1	1	1	1	1	1	1	1
dl-methionine	0.17	0.17	0.20	0.12	0.15	0.21	0.22	0.15	0.18	0.16
l-lysine	0.19	0.15	0.16	0.22	0.20	0.11	0.14	0.12	0.14	0.18
l-tryptophane		0.01								
l-threonine				0.005	0.02					
Nutritional values^2^										
ME calculated (kcal/kg)	2601	2600	2901	2303	2905	2302	2599	2597	2903	2302
CP calculated (g/kg)	16.5	16.6	19.5	13.5	13.5	19.4	19.6	13.6	16.4	16.5
Lysine dig. (%)	0.72	0.72	0.85	0.59	0.59	0.85	0.86	0.60	0.71	0.72
Methionine dig. (%)	0.42	0.42	0.49	0.34	0.34	0.51	0.50	0.34	0.42	0.42
Threonine dig. (%)	0.51	0.53	0.62	0.418	0.42	0.64	0.62	0.42	0.52	0.53
Tryptophan dig. (%)	0.17	0.16	0.19	0.13	0.13	0.19	0.20	0.13	0.16	0.16
Calcium (%)	1.2	6.6	0.6	6.6	0.5	6.6	0.6	6.6	0.6	6.6
Total P (%)	0.65	0.52	0.59	0.59	0.56	0.62	0.67	0.50	0.59	0.60
Available P (%)	0.32	0.32	0.32	0.32	0.32	0.32	0.32	0.32	0.32	0.32
Coarse particles >2.5 mm	19.3	26.3	31	16.5	29	21.6	22.2	22.5	28.3	22
Fine particles<1.18 mm	48.7	28.9	28.5	52.6	31.2	44	37.9	32.2	26.7	35.2

1 Premix: supplied per kg of diet at vitamin A 750 000 UI; vitamin D_3_ 30 0000 UI; vitamin E (tocopherol acetate 3a700) 1000 UI; vitamin K3 100 mg; Zinc 10 000 mg; manganese 10 000 mg; copper 1000 mg; iron 4000 mg; iode 150.0 mg; selenium 30.0 mg; canthaxanthin 1950.0 mg; β-apocarotenoïde acid 2000.0 mg; lutein 3560.0 mg; zeaxanthin 200.0 mg; cryptoxanthin 80.0 mg; citric acid 400.00 mg; orthophosphoric acid 660.00 mg; ethoxyquin 400 mg; and propyl gallate (E310) 400 mg.

2 Nutritional values were calculated out of Sauvant *et al.* (2004).

### Measurements

Particle size profile was measured. Four different sieves were used to characterize the particle size (2.5, 1.6, 1.18 and >1.18 mm). Feed intake was individually recorded every week for the morning and afternoon fractions. Egg production was recorded on a daily basis. Egg weight was recorded twice a week by weighing all eggs produced on the measuring day. Weights of egg components (yolk, albumen and shell) were determined on the daily samples every 4 weeks, starting at week 22. Shells were washed and dried for 12 h in a drying oven at 70°C and then weighed.

BW was recorded at 16, 19, 23, 27, 31, 35 and 39 weeks of age. Body composition was predicted with a noninvasive method based on bioelectrical impedance analysis at 19, 27 and 35 weeks of age. The technique has been developed by the company INZO°^1^ and predicts the body composition by an instrument of impedance, which was calibrated through the chemical analysis of birds. Impedance measurements of resistance (Rs) and reactance (Xc) are taken using two electrodes placed on the animal’s legs and distributing a very low-intensity current. It is assumed that the electrical impedance of the biological organism is indicative of the highly conductive fat-free component (Berg and Marchello, 1994). The electrical impedance of a tissue depends on its fluid and electrolyte content (Rutter *et al*., 1998) and therefore on the electrical properties of different tissues in connection with their relative proportions of muscle, lipid, water and ash. For the duration of a full day, in week 27, feed intake kinetics of hens was measured in the second room. Every hour, the feeders were weighed to set the amount of food ingested by the hens during this period. This operation was performed for the two fractions (morning and afternoon) throughout the light period from 0400 to 2000 h. Metabolizable energy (ME; kcal/bird per day) and protein (g/bird per day) intake were calculated as a product of feed intake and ME and protein contents of the experimental diets, respectively.

At the end of the trial, 10 birds per treatment in the second room were randomly selected, weighed and injected with Na pentobarbital solution (1 ml/kg) for killing birds without pain. The weight of the digestive organs was recorded to assess the effect of feeding system on these organs. The abdominal cavity was opened and the digestive tract dissected and separated into the gizzard, small intestine, pancreas and liver. The small intestine was separated into the duodenum, jejunum and ileum. Each segment was first emptied before weighing. The gizzard was placed into an iced container (−4°C) for 24 h to facilitate the removal of the surrounding fat before being emptied and weighed.

For the evaluation of the apparent digestibility of the hens, Titanium dioxide was added at a rate of 5 g/kg as a dietary marker in the two fractions. Chemical analyses were performed for TiO_2_ concentrations in each fraction of the diet and in the feces. Analysis of TiO_2_ was performed by colorimetric method. In the presence of sulfuric acid, TiO_2_ and H_2_O_2_ cause a yellow coloration read by absorbance at 410 nm (TECAN, Infinite200, Grödig, Austria). This method was developed by Short *et al.* (1996). Apparent digestibility was calculated as follows:
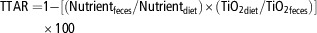
where TTAR is total tract apparent retention, Nutrient_feces_ and TiO_2feces_ are the concentrations of dietary components and of TiO_2_ in the excreta, Nutrient_diet_ and TiO_2diet_ represent the concentrations of the same dietary components and the TiO_2_ in the feeds. Concentrations in the sequential diets were obtained by combining the actual quantities of ingested morning and afternoon fractions with their respective content of nutrient and titanium dioxide. At 32 weeks of age, blood sampling in the wing vein was used to define the level of hematocrit as an indicator of the effect of red mites on the health of the hens.

### Statistical analysis

Data collected during the experiment were analyzed based on two different periods: during the increase of lay (from weeks 20 to 26) and after the peak (from weeks 27 to 39). Averaged values from each period were analyzed using StatView (version 5, SAS Institute Inc., Cary, NC, USA). A two-way ANOVA was used to test treatment and room effect on all measured parameters. If the ANOVA was significant (*P*<0.01: **, *P*<0.05: *, *P*<0.1: obtained *P*-value, *P*>0.1: ns), a *post hoc* Bonferroni test was used, differences between diets on the same line are illustrated with different letters (a, b, …) if *P*<0.05, and with capital letters (A, B, …) if *P*<0.1.

## Results

### Diet and room effect on egg production, BW, body composition and FCR

#### Weeks 20 to 26

(Table 2) No difference owing to the diet was observed for the laying performances (egg weight, egg production and egg mass) and for the FCR. No difference was observed for initial BW and body composition at week 19. BW gain (BWG; weeks 20 to 27) was significantly affected by the diets (*P*=0.02). Hens with E+P+/E−P− had lower gain than E+P−/E−P+. There was a difference between the two rooms for the egg mass (*P*=0.01) and a tendency for egg production (*P*=0.06). An interaction between the diet and room was observed (*P*=0.04). Hens consuming E0P0/E0P0 in the second room having higher BWG (78.2 g) than those in the first room (60.2 g), whereas for all other birds hens in the second room had lower BWG than those of the first one.

**Table 2:** Effects on egg production, FCR, BW and body composition of laying hens from 20 to 39 weeks old offered a combination of 62.5 g of morning fraction between 0830 and 1530 h and 62.5 g of afternoon fraction between 1530 and 0830 h (no light between 2000 and 0400 h)

	E0P0/E0P0	E+P+/E−P−	E+P−/E−P+	E0P+/E0P−	E+P0/E−P0	Room 1	Room 2	s.e.m.	Diet^1^	Room^1^	Diet×room^1^
Weeks 20 to 26											
Egg production (%)	82.4	86	80.7	79.3	82.8	84.9	80.3	1.2	ns	0.06	ns
Egg weight (g)	53.4	50.9	51	53.7	53.8	52.6	52.6	0.7	ns	ns	ns
Egg mass (g/hen per day)	43.1	43.8	41.1	41.9	43.8	44.3	41.6	0.6	ns	*	ns
FCR	2.42	2.36	2.54	2.68	2.42	2.45	2.51	0.05	ns	ns	ns
Initial BW week 19 (g)	1421	1419	1394	1427	1412	1424	1408	8	ns	ns	ns
BW gain (g)	272ab	216a	283b	260ab	273ab	282	245	7	*	**	*
Body composition: % fat week 19	11.4	11.3	11	11.6	11.2	11.3	11.4	1.4	ns	ns	ns
Body composition: % protein week 19	18.3	18.3	18.3	18.2	18.3	18.3	18.2	0.3	ns	ns	ns
Weeks 27 to 39											
Egg production (%)	98.1	96.8	98.1	95.9	95.4	98.3	95.8	0.3	ns	***	ns
Egg weight (g)	58	57	57.7	58.6	58.9	59.3	57.2	0.3	ns	***	ns
Egg mass (g/hen per day)	56.9	55.2	56.7a	56.2	56.2	58.2	54.8	0.4	ns	***	ns
FCR	2.04	2.11	2.07	2.07	2.06	2.03	2.09	0.03	ns	**	ns
BW week 27 (g)	1692	1635	1677	1687	1685	1706	1653	9	ns	**	ns
Final BW week 39 (g)	1760	1699	1761	1733	1695	1787	1686	12	ns	**	ns
BW gain (g)	68b	64	81a	45	9b	81	32	8	*	***	ns
Body composition: % fat week 27	16.1	15.2	16	16	16.1	16.3	15.6	0.2	ns	*	ns
Body composition: % protein week 27	17.3	17.4	17.3	17.3	17.3	17.3	17.3	0.3	ns	ns	ns
Body composition: % fat week 35	16.8	16.5	16.6	16.9	16.4	17.6	15.9	0.2	ns	***	ns
Body composition: % protein week 35	17	17	17.1	17	17.1	16.9	17.2	0.4	ns	***	ns

FCR=feed conversion ratio (feed intake/egg mass). Fractions were a combination of energy (E) and protein (P) levels, with low (−), medium (0) or high (+) values. *P*>0.05; values within a row with common letters (a, b) differ significantly using Bonferroni–Dunnet test at 5% significance level.

1 **P<0.01; *P<0.05.

#### Weeks 27 to 39

Egg production, egg weight, egg mass, BW (weeks 27 and 39), BWG and FCR were reduced for hens in the second room compared with hens in the first one. No diet effect was observed on egg performance (egg production, egg weight and egg mass), FCR and BW at 39 weeks of age. The only significant difference is for hens with E+P0/E−P0, which had lower BWG than hens with E+P−/E−P+ (no difference with the other diets). There was no interaction between room and diet for all measured parameters. There was neither a diet nor room effect for the egg components. For body proportion in protein and fat, an effect of age was observed. There was no diet effect on fat and protein compositions on weeks 19, 27 and 35.

After hens’ slaughtering at 39 weeks of age, the main digestive organs and abdominal fat were weighed. However, there were no significant differences between diets on any tested values (data not shown). There was no diet effect on the apparent digestibility. The values of apparent digestibility performed on sequentially fed hens were similar to values usually obtained on continuously fed hens, as observed in previous studies with similar bred hens. Hematocrit on week 32 underlined a difference between the two rooms: 30.5% in the first room compared with 25.4% in the second one.

### Diet and room effects on feed intake, ME intake and CP intake

#### Weeks 20 to 26

(Table 3) All parameters were degraded in the second room. There was no diet effect on total and afternoon feed intakes. However, diet affected morning feed intakes (with a tendency for lower level for E0P+/E0P− and E0P0/E0P0 compared with E+P−/E−P+). Diets did not affect total ME intake, but hens had different ME intake level during the day. For the morning ME intake, all birds with high energy in the morning (E+P+/E−P−, E+P−/E−P+ and E+P0/E−P0) ate more energy compared with birds with lower levels on energy in the morning (E0P+/E0P− and E0P0/E0P0). Concerning the afternoon ME intake, hens with an E− fraction in the afternoon ate less energy than hens with the two other diets (E0P+/E0P− and E0P0/E0P0). For total CP intake, no diet effects were observed, but diets affected the CP intake during the day. For the morning CP intake, three different groups were observed. Hens with P+ in the fraction of the morning (E+P+/E−P− and E0P+/E0P−) had the highest level of CP intake, hens with P0 in these fractions in the morning (E0P0/E0P0 and E+P0/E−P0) had an intermediate level of CP intake and hens with P− in the morning (E+P−/E−P+) had the lowest level of CP intake.

**Table 3:** Effects on feed, ME and CP intakes of laying hens from 20 to 39 weeks old offered a combination of 62.5 g of morning fraction between 0830 and 1530 h and 62.5 g of afternoon fraction between 1530 and 0830 h (no light between 2000 and 0400 h)

	E0P0/E0P0	E+P+/E−P−	E+P−/E−P+	E0P+/E0P−	E+P0/E−P0	Room 1	Room 2	s.e.m.	Diet^1^	Room^1^	Diet×room^1^
Weeks 20 to 26											
Total feed intake (g/bird per day)	102.8	102.3	103	103.1	104.9	106	101.1	0.8	ns	**	ns
Morning	48.3b	49.5ab	52a	47.7b	50.7ab	50.8	48.8	0.4	**	**	ns
Afternoon	54.5	52.8	51	55.4	54.2	55.2	52.4	0.5	ns	**	ns
Total ME intake (kcal/bird per day)	267	265	268	268	272	275	263	2	ns	**	ns
Morning	126b	143a	151a	124b	147a	142	136	2	***	**	ns
Afternoon	141b	122a	117a	144b	125a	134	127	2	*	**	ns
Total CP intake (g/bird per day)	17	16.7	16.9	16.9	17.2	17.4	16.6	0.1	ns	**	ns
Morning	7.9c	9.6a	7b	9.3a	8.3c	8.6	8.3	0.1	***	*	ns
Afternoon	9.1c	7.1a	9.9b	7.6a	8.9c	8.8	8.3	0.1	***	**	ns
Weeks 27 to 39											
Total feed intake (g/bird per day)	115.7	116.1	116.8	115.8	115.4	118.2	114.3	0.6	ns	**	ns
Morning	53.4	53.8	54.5	51.8	53.6	54	53	0.4	ns	ns	*
Afternoon	62.3	62.3	62.3	64	61.8	64.2	61.3	0.4	ns	***	ns
Total ME intake (kcal/bird per day)	301	300	302	301	298	306	296	2	ns	**	ns
Morning	139b	156a	156a	135b	156a	150	147	1	***	0.06	*
Afternoon	162b	144a	146a	166b	142a	156	149	1	**	***	ns
Total CP intake (g/bird per day)	19.1	18.9	19.4	18.8	19	19.4	18.8	0.1	ns	**	ns
Morning	10.8a	10.5a	7.3b	10.1a	8.8c	9.2	9	0.1	***	ns	0.06
Afternoon	8.3a	8.4a	12.1b	8.7a	10.2c	10.2	9.8	0.1	***	**	ns

Fractions were a combination of energy (E) and protein (P) levels, with low (−), medium (0) or high (+) values. *P>*0.05; values within a row with common letters (a, b, c) differ significantly using Bonferroni–Dunnet test at 5% significance level.

1 **P<0.01; *P<0.05.

#### Weeks 27 to 39

**Figure 1:**
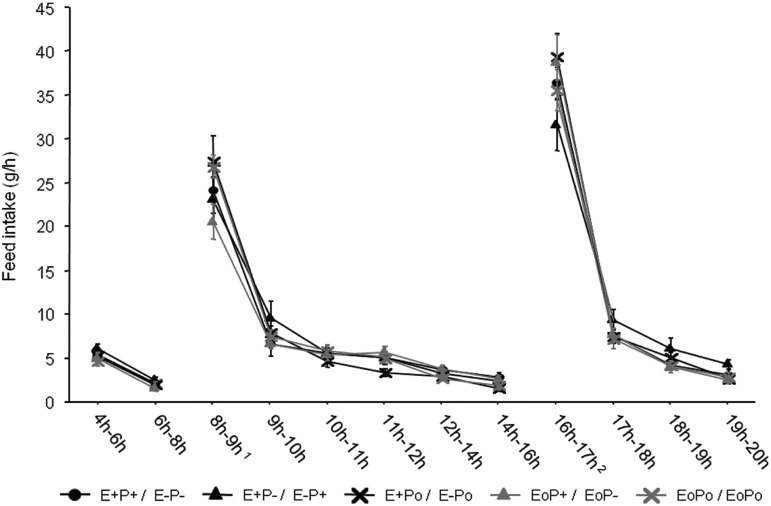
Effects of different diets on feed intake (g/h) during a whole day at 27 weeks old for laying hens fed between 19 and 39 weeks of age, with a combination of 62.5 g of morning fraction between 0830 and 1530 h and 62.5 g of afternoon fraction between 1530 and 0830 h (no light between 2000 and 0400 h). ^1^Morning distribution; ^2^afternoon distribution.

All parameters were degraded in the second room. There was no diet effect on feed intakes and total ME intake. For ME intake in the morning, all hens with high energy in the morning (E+P+/E−P−, E+P−/E−P+ and E+P0/E−P0) ate more energy compared with birds with lower level on energy in the morning (E0P+/E0P− and E0P0/E0P0). For the afternoon ME intake, all birds with an E− fraction in the afternoon (E+P+/E−P−, E+P−/E−P+ and E+P0/E−P0) ate less energy than hens with the two other diets (E0P+/E0P− and E0P0/E0P0). Concerning total CP intake, there was no difference between all diets. For the morning CP intake, three different levels were observed: a high level of protein intake for E+P+/E−P−, E0P+/E0P− and E0P0/E0P0, an intermediate level for E+P0/E−P0 and the lowest level for E+P−/E−P+. For CP afternoon intake, three levels were still reported: the highest for E+P−/E−P+, an intermediate one for E+P0/E−P0 and a low level for E+P+/E−P−, E0P0/E0P0 and E0P+/E0P−. Concerning feed intake dynamics (Figure 1), there was no diet effect on the eating rate. However, this figure allows us to highlight the daily shape of ingestion induced by SF: feed intake was induced by the diet distribution, and hens were expecting the next diet because of the learning of the timetable of the feed supply.

## Discussion

The main objective of this work was to study the influence of different kinetics of nutrient supply in SF on performances of laying hens. Only sequential diets were given. One treatment brought E0P0 in the morning and E0P0 with Ca in the afternoon, which may mimic a continuous diet regarding energy and protein supply. However, the two fractions were contrasted in their raw materials content and their particle size. These differences might explain that for the 20- to 26-week period, hens with E0P0 in the morning (19% of large particles and 49% fines) consumed less than hens that had E0P0+Ca at the same time (29% of large particles and 31% fine). Indeed, coarse particle induces an increase in intake compared with higher percentage of fine particles diet (Nir *et al.*, 1990; Safaa *et al.*, 2009). For the first room, with a low environmental pressure, rate of lay and egg mass were similar to those observed in SF in previous studies (Umar Faruk *et al.*, 2010; Traineau *et al.*, 2013). However, FCR (+5.1%) and egg weight (−5.3%) were different from those observed by Traineau *et al.*, (2013), which may be because of a difference in total protein intake 17.5 g/day in our trial compared with 19.0 g/day for Traineau *et al.* (2013). Summers and Leeson (1985) showed that egg size could be altered by 1.3 g when the protein intake varied between 17 and 18.9 g.

For the duration of the experiment, environmental pressure increased in the second room. From weeks 20 to 26, feed intake levels were lower in the second room compared with the first one, whereas egg weight, weight gain and FCR were not affected. From weeks 27 to 39, difference between the two rooms strongly increased. Red mites’ pressure can lead to decreases in feed consumption and reduce eggs’ quality and production (Chauve, 1998; Lesna *et al.*, 2009). To implement the evidence of the negative effect of these red mites, measure of hematocrit on week 32 showed a strong difference between the two rooms (30.5% in the first room compared with 25.4% in the second one). However, the ranking between the diets was similar in the two rooms, indicating that sequential diets were impacted in the same order of magnitude regardless of their contents.

The main question was to determine the impact of nutrient distribution on hens’ responses. First of all, regardless of the protein or energy timetable supply, a higher intake was observed for the afternoon fraction compared with the morning one as obtained by Umar Faruk *et al.* (2010) and Traineau *et al.* (2013). Despite contrasted energy and protein supply timetables, hens were not affected on the quantity of feed intake. The morning and afternoon fractions differed on the energy and protein level between all diets. However, hens did not seem to be able to fit their intake level on the diets’ composition. Some authors showed that hens are not able to fit their feed intake on CP level in the diet (Summers and Leeson, 1985). These results are in accordance with our observation on the nonregulation of feed intake on protein content of the diet. On the contrary, regarding the effect of the energy level, hens seem to be able to regulate their feed intake on ME level of the diet (Plavnik *et al.*, 1997; Nahashon *et al.*, 2005). Bouvarel *et al.* (2010) showed that hens reduced their feed intake with the increase in energy content in the diet. In our studies, hens did not seem to be able to adjust their consumption on the energy density of the offered fraction. However, energy and protein intakes’ dynamics are different during the day between diet, keeping in mind that the afternoon fraction was not energy rich (no E+).

The absence of differences with contrasted fractions does not allow to define clearly the nutritional needs in the different time of day in SF. It might be proposed that if hens were offered enough nutrients in the day, their sequence did not seem to have a strong impact on the birds’ performances. In this study, as hens received in the morning a fraction rich in energy (or an average level), it might suggest that in SF, it would be better to bring the fraction rich in energy in the morning to get satisfactory results. A low level in the energy fraction in the morning may not be possible because of the low level of feed intake of this fraction, which is constantly observed during all work in SF. These studies have also demonstrated that, despite significant health pressure, hens fed sequentially are able to produce eggs and ensure a normal growth.

## Footnotes

1
INZO° SAS rue de l’Eglise-BP 50019 02407 Chierry Cedex.
